# A potential target gene CD63 for different degrees of intervertebral disc degeneration

**DOI:** 10.1038/s41598-022-05021-4

**Published:** 2022-01-19

**Authors:** Sheng Gao, Shu Jia, Xutao Fan, Chengcan Gao, Qingwei Li, Yuxue Wu, Chunyang Meng

**Affiliations:** 1 Department of Medicine, Qingdao University, Qingdao, China; 2 Clinical Research Team of Spine and Spinal Cord Diseases, Medical Research Center, Affiliated Hospital of Jining Medical University, Jining, China; 3 Department of Spine Surgery, Affiliated Hospital of Jining Medical University, 89 Guhuai Road, Jining, Shandong China

**Keywords:** Computational biology and bioinformatics, Biomarkers, Medical research

## Abstract

Understanding molecular mechanisms of intervertebral disc degeneration (IDD) and providing a novel target for the treatment of IDD have important implications. We sought to explore a new promising gene target for the treatment of IDD. This study integrated 19,678 genes of 38 IDD patients from two gene datasets. Differentially Expressed Genes (DEGs) of annulus fibrosus were analyzed in groups with mild disc degeneration (MDD) and severe disc degeneration (SDD). We screened the hub gene through biological information technology (bioinformatic) methods. Then, we further validated the hub gene using annulus fibrosus and nucleus pulposus tissues from 12 patients with qRT-PCR. In addition, we explored its underlying molecular mechanism with GO, KEGG and GSEA. Through multiple screening bioinformatics methods, the hub gene CD63 was identified. The qRT-PCR explored that CD63 decreased significantly in SDD group compared to that in MDD group (P < 0.001). The GO, KEGG and GSEA of CD63 explored significant enrichment of the molecular features (P < 0.001), including the cellular component (Extracellular matrix, P < 0.001), the molecular function (collagen binding, P < 0.001), the biological processes (protein targeting, collagen fibril organization and platelet degranulation, P < 0.001) and the signaling pathways. Our research explored and validated a new regulatory gene, CD63 for different degrees of IDD. A new novel form of therapeutic target for IDD may be developed.

## Introduction

Low back pain (LBP) is a common cause of disability and it negatively effects on the quality of life of patients globally^1^. However, the commonly reported and targeted factor for intervention is intervertebral disc degeneration (IDD). IDD plays a significant role in LBP and associates strongly with dysfunction and structural breakdown of intervertebral disc (IVD)^2^. The IDD is currently recognized among the common causes of morbidity^2,3^. Nonetheless, the etiology of IDD is multifactorial and their complex mechanisms are not well understood. Some studies have reported factors including apoptosis, insufficient nutritional supply and excessive mechanical load^4^. Currently, treatment for IDD is largely dependent on surgical intervention with disc excision and spinal fusion, for late-stage IDD as well as symptomatic relief. In the early stages for example, conservative treatments like bed rest, painkillers, or physiotherapy are usually the preferred option^5^. When it seriously affects the quality of people’s life, treatment for IDD is largely dependent on surgical intervention with disc excision and spinal fusion^6^. It is important to note that surgical treatment does not preserve the function of disc. The pathogenesis of IDD has been vastly linked to a lot of factors for instance, spine injuries, aging, spine deformities and genes^7,8^. A few studies have reported on numbers of genes and how they correlate with functional and structural changes within the IVD^9–12^. Even though such studies shed light on molecular aspect of IDD, there is still little knowledge regarding gene factors and their contribution to pathogenesis of IDD as well as therapeutic targets related to the disease. Determining the onset and different degrees of intervertebral disc degeneration of IDD based on relevant regulatory genes is an important insight for the prevention of IDD and more effective treatment options for the disease. This will enable the development of novel therapies for IDD that are more specific. Therefore, it was necessary to carry out this study. It may help us further understand the molecular mechanism of IDD and provide new promising targets for the diagnosis and treatment of IDD. So, we sought to explore a new promising gene target for the treatment of IDD.

## Materials and METHODS

### Data collection and preparation

We collected a total of 19,678 genes from 38 degenerative disc patients with complete clinical information from Gene Expression Omnibus (GEO) database for external analysis. Samples from two datasets GSE15227 and GSE23130 were selected. According to the Pfirrmann classification of IDD^13^, the gene expression profiles were obtained from 15 samples of GSE15227 with 5 mild disc degeneration (MDD) (grade I–II) and 10 severe disc degeneration (SDD) (grade III–V). Among the 23 samples data in GSE23130, gene expression profiles were obtained from 6 MDD (grade I–II) and 17 SDD (grade III-V). Both the two datasets were based on GLP1352 detection platform and come from the same microarray [U133_X3P] Affymetrix Human X3P Array.

The R package “limma” was used to transform RNA expression data from a biased distribution to an approximate normal distribution. The ComBat function of package “sva” was used to remove the batch effect of samples in datasets. The gene expression profiles data was standardized with log2 logarithm and package “limma” to eliminate the heterogeneity. A total of 19,678 genes were obtained through normalization of the two datasets.

### Differential analysis of genes

A total of 11 samples in the MDD group and 27 samples in the SDD group were analyzed. The R package “limma” was used to calculate the P-values and Fold Change (FC) of gene expression differences between the MDD group and SDD group. The P < 0.05 and |log2 FC|> 1 were selected as screening threshold for significant differential genes (DEGs). The filtered values of DEGs were extracted from data of standardized expression profiles. We also compared the similarities of the two datasets (GSE15227 vs. GSE23130) and performed a heatmap of the differential gene expression profiles of MDD group and SDD group in the two datasets.

### Weighted correlation network analysis for DEGs

Weighted Correlation Network Analysis (WGCNA) of DEGs was carried out to further describe the association patterns of gene expression profiles. We calculated the Pearson correlation of DEGs with package “WGCNA”. The adjacent matrix was transformed into a topological overlap matrix. Thereafter, we calculated the dissimilarity of gene sets and gained the hierarchical clustering tree of genes with R, which were cut into different modules with a minimum number of 10 module genes. To screen out the hub module of IDD, correlations between gene modules and the grades of degenerative IVD samples were analyzed. The R package “WGCNA” was used to calculate Pearson Correlation (Cor) and P-values between DEGs and Pfirrmann classification of IDD with the screening criteria of Cor > 0.6 and P < 0.01. The key genes (Gene set A) that were closely related to IDD were identified and documented.

### Construction of gene co-expression network

Cytoscape package “CytoNCA” were used to analyze three topological characteristics of each node in the network. This included degree of the node, number centricity and proximity to centrality. We extracted top ten common genes in each topological feature and constructed a co-expression genes (Gene set B) network with Cytoscape.

### Select the target gene

We took an intersection of the two key gene sets that screened by WGCNA (Gene set A) and Co-expression Network (Gene set B), and having the target gene CD63. To further estimate whether CD63 was closely related to IDD, we analyzed the Pearson Correlation between CD63 and IL1^14^, ECM^15^, COL2^16^, TIMP^17^, MMP^18,19^ as well as ADAMTS^19,20^ which were closely related to IDD significantly.

### GO and KEGG analysis of gene set for hub module

To further explore the mechanism of intervertebral disc degression, we performed Gene Ontology (GO) and Kyoto Encyclopedia of Genes and Genomes (KEGG)^21–23^ gene-set enrichment analysis of the hub modules in WGCNA. The GO defines gene function from three aspects: cellular component (CC), molecular function (MF) and biological process (BP). The KEGG provide enrichment analysis of gene pathway. The DAVID was used to perform GO and KEGG pathway enrichment analysis for the hub module. Then, we screened CC, BP, MF and signaling pathway with differential expression in the hub module of IDD. To fully understand the GO enrichment results associated with IDD, R was used to visualize the gene-set enrichment results.

### Gene Set enrichment analysis

The GSEA were used to further identify the significantly enrichment function. The reference gene set used in this study were c5.all.v7.1.symbols.gmt and c2.cp.kegg.v7.1.symbols.gmt, with nominal P < 0.05 and FDR < 0.25 used as threshold to screen significant enrichment functions and pathways. Enrichment functions and signaling pathways of CD63 obtained in GSEA were intersected with the GO and KEGG pathways that were obtained from hub module of IDD.

### Patient tissue samples

To further study and validate the role of CD63 in different degrees of IDD, annulus fibrosus (AF) and nucleus pulposus (NP) tissues from 12 patients (Table 1) were used to measure the expression of CD63 with qRT-PCR. The inclusion and exclusion criteria were as follows. Inclusion criteria: patients with mild and severe lumbar disc degeneration and treated with discectomy. Exclusion criteria: patients with intervertebral disc calcification and acute infection. The experimental protocols were approved by the Ethics Committee of Affiliated Hospital of Jining Medical University (Jining, China). Before the experiments, tissue samples of all the patients were collected with informed consent. This study performed in accordance with the declaration of Helsinki and guidelines of the Ethics Committee of Affiliated Hospital of Jining Medical University. The clinical samples were collected from patients with lumbar discectomy and were divided into MDD group (n = 6) (Supplementary Fig. 1) and SSD group (n = 6) (Supplementary Fig. 2) according to Pfirrmann classification^13^. In SDD of grade III-V, the boundary between annulus fibrosus and annulus fibrosus disappears, as well as the height of intervertebral space decreases, however, MDD of grade I and II only with signal intensity changes in MRI.

**Table 1:** Clinical characteristics of patients.

Item	MDD group (n = 6)	SDD group (n = 6)	P
Age (years)	33	58	
	55	38	
	39	50	
	40	38	
	41	31	
	35	44	
Age(M ± SD)	40.50 ± 7.7395	43.17 ± 9.6833	0.6097
**Clinical feature**			
Sex (male)	4	3	> 0.999
Low back pain	5	4	
Lower extremity radicular pain	6	6	

### Total RNA isolation and qRT-PCR

Total RNA was extracted respectively from AF and NP tissues of 12 patients with TRIzol Reagent (Invitrogen Life Technologies, Carlsbad, CA, USA). Reverse transcribed using PrimeScript RT Master Mix (Invitrogen Life Technologies, Carlsbad, CA, USA). qRT-PCR was performed using cDNA and SYBR mixture (CWBio, Beijing, China) to quantify the mRNA expression levels of CD63 and normalized it against β-actin. Relative expression levels of mRNA were computed with the method of 2^−ΔΔ Ct^.

### Statistical analysis

The statistical analyses were calculated by GraphPad Prism 8. Significance was established at p < 0.05 and processed with GraphPad Prism 8.

### Ethics declarations

We declare all the tissue samples of patients were collected with informed consent. The patients and their families were informed that data from the cases would be submitted for publication, and gave their consent. The experimental protocols were approved by the Ethics Committee of Affiliated Hospital of Jining Medical University (Jining, China). This study performed in accordance with the declaration of Helsinki and guidelines of the Ethics Committee of Affiliated Hospital of Jining Medical University.

## Results

### Hub module and genes

**Figure 1:**
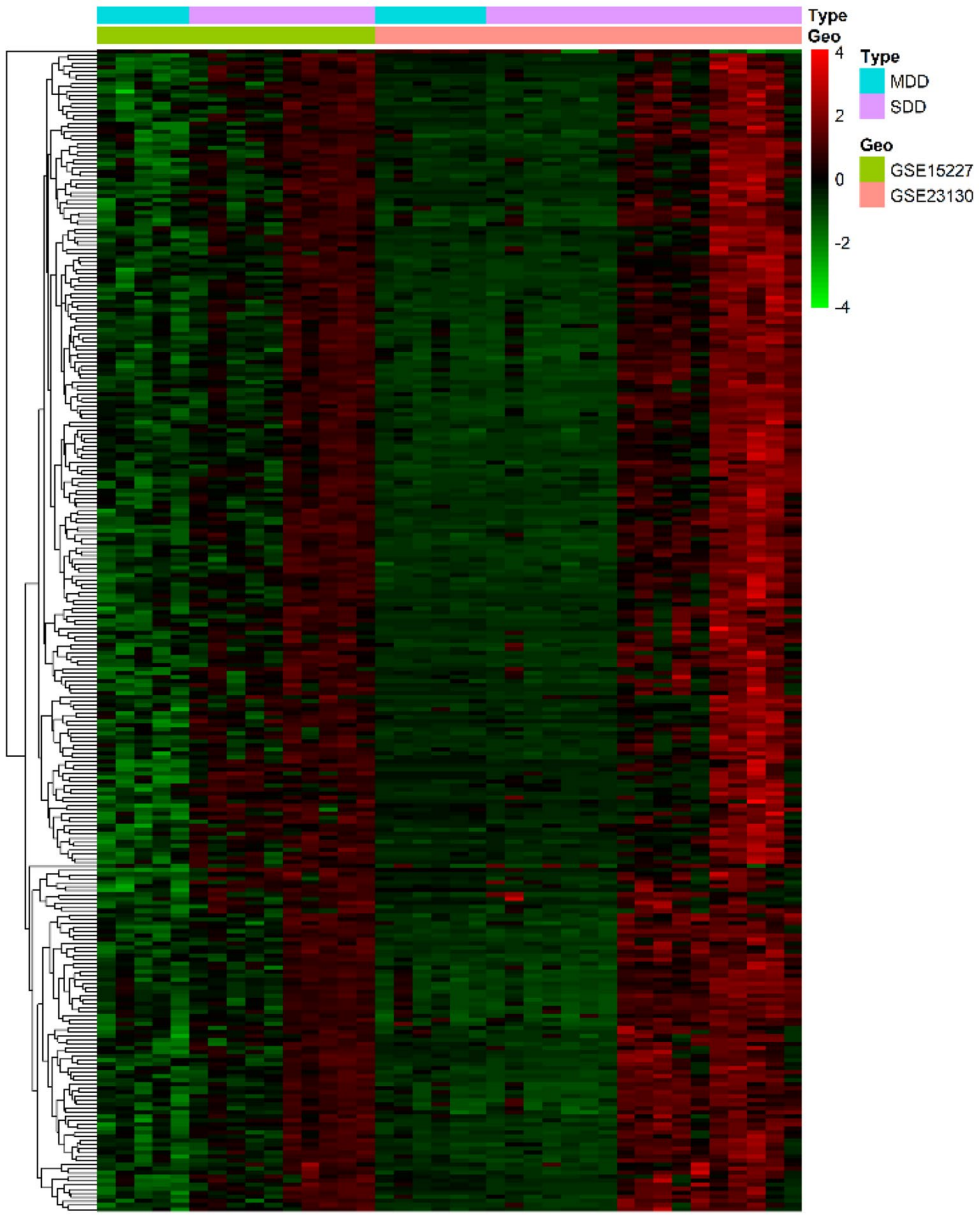
Heatmap of differential gene expression profiles in GSE15227 and GSE23130. The heatmap showed the similarity of the two datasets in SDD group and MDD group. Red represents up-regulated genes and green represents down-regulated genes.

**Figure 2:**
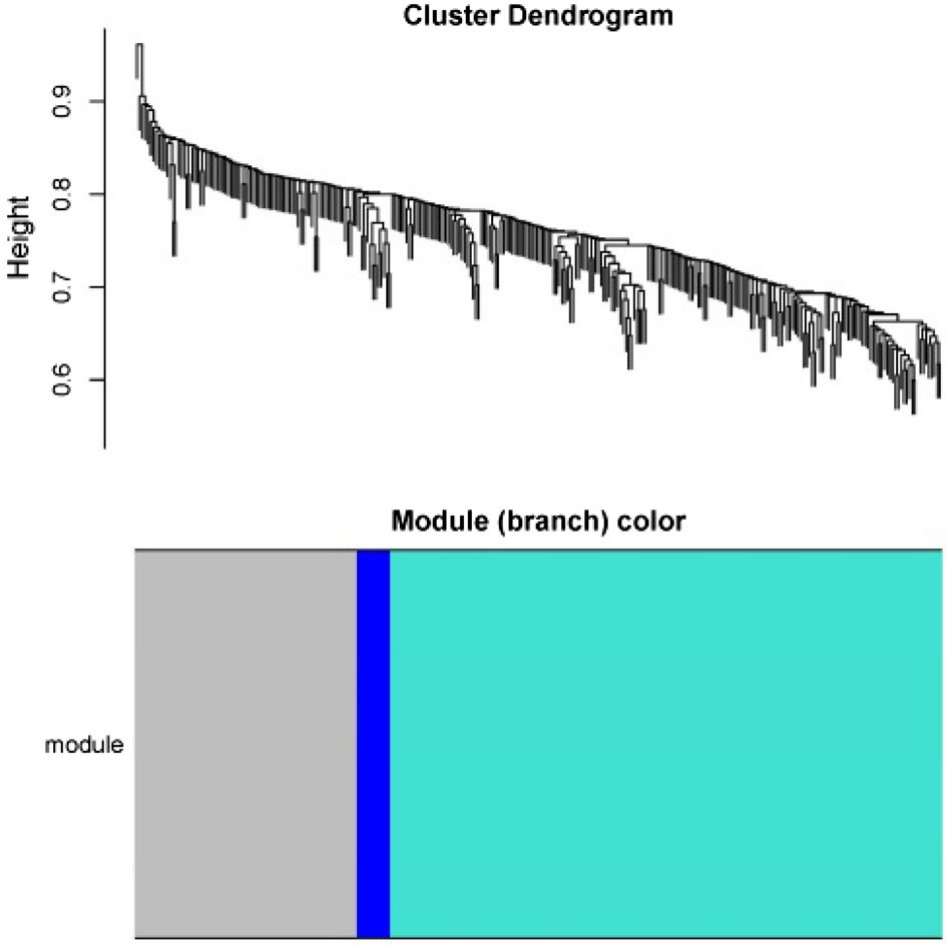
Gene modules. Three gene modules of MEturquoise, MEblue and MEblue were obtained. The MEturquoise is the hub module.

Based on the threshold of P < 0.01 and |log2 FC|> 1, 290 differential genes were screened out from the two datasets. The heatmap of the expression profiles of DEGs showed the similarity of the two datasets in SDD group and MDD group (Fig. 1). Three gene modules of MEturquoise, MEblue and MEblue were obtained from the hierarchical clustering of WGCNA (Fig. 2). Among these modules, MEturquoise had 198 genes, with the most significant difference of Cor = 0.5659 and P = 0.000213 (Table 2). In the Pearson correlation analysis between DEGs and IDD, nine key genes were screened (Cor > 0.6 and P < 0.001) (Table 3).

**Table 2:** The Pearson Correlation and P-values between gene modules and IDD.

Gene module	Cor	P
MEblue	0.4744	0.002621
MEturquoise	0.5659	0.000213
MEgrey	0.4861	0.001976

**Table 3:** The Pearson Correlation and P-values between DEGs and IDD.

DEGs	Cor	P
C2CD2	0.6501	< 0.001
GSTP1	0.6343	< 0.001
SGK1	0.6318	< 0.001
CD63	0.6222	< 0.001
LUM	0.6171	< 0.001
SCRG1	0.6131	< 0.001
C1S	0.6074	< 0.001
REEP5	0.6034	< 0.001
CLIC1	0.6016	< 0.001

### Gene co-expression network

**Figure 3:**
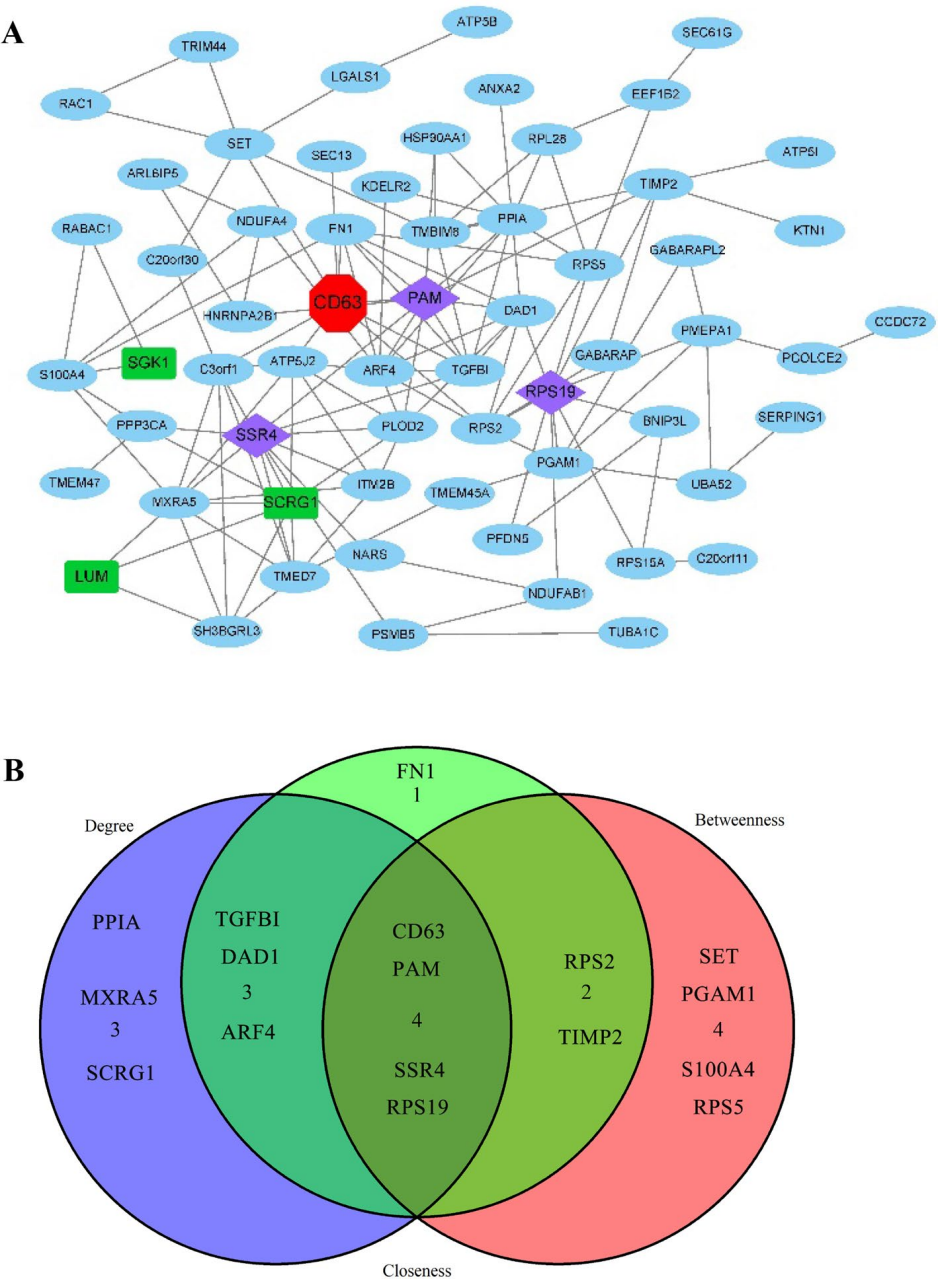
Gene co-expression network and topological features. (**A**) A total of 81 gene nodes and 43 interaction pairs obtained in the Weighted Correlation Network Analysis with the threshold of Cor > 0.6. (**B**) Intersection of the top 10 genes in each Topological Features (Betweenness, Closeness and Degree) related to intervertebral disc degeneration.

Through the analysis of WGCNA, a total of 81 gene nodes and 43 interaction pairs were obtained with the threshold of Cor > 0.6 as explored in the Gene Co-expression Network (Fig. 3A). It was found that the top 10 genes related to IDD in each Topological Features (Degrees, Meso-centricity, Proximity-centricity) including CD63, PAM, SSR4 and RPS19 (Fig. 3B).

### Selection of target genes

**Figure 4:**
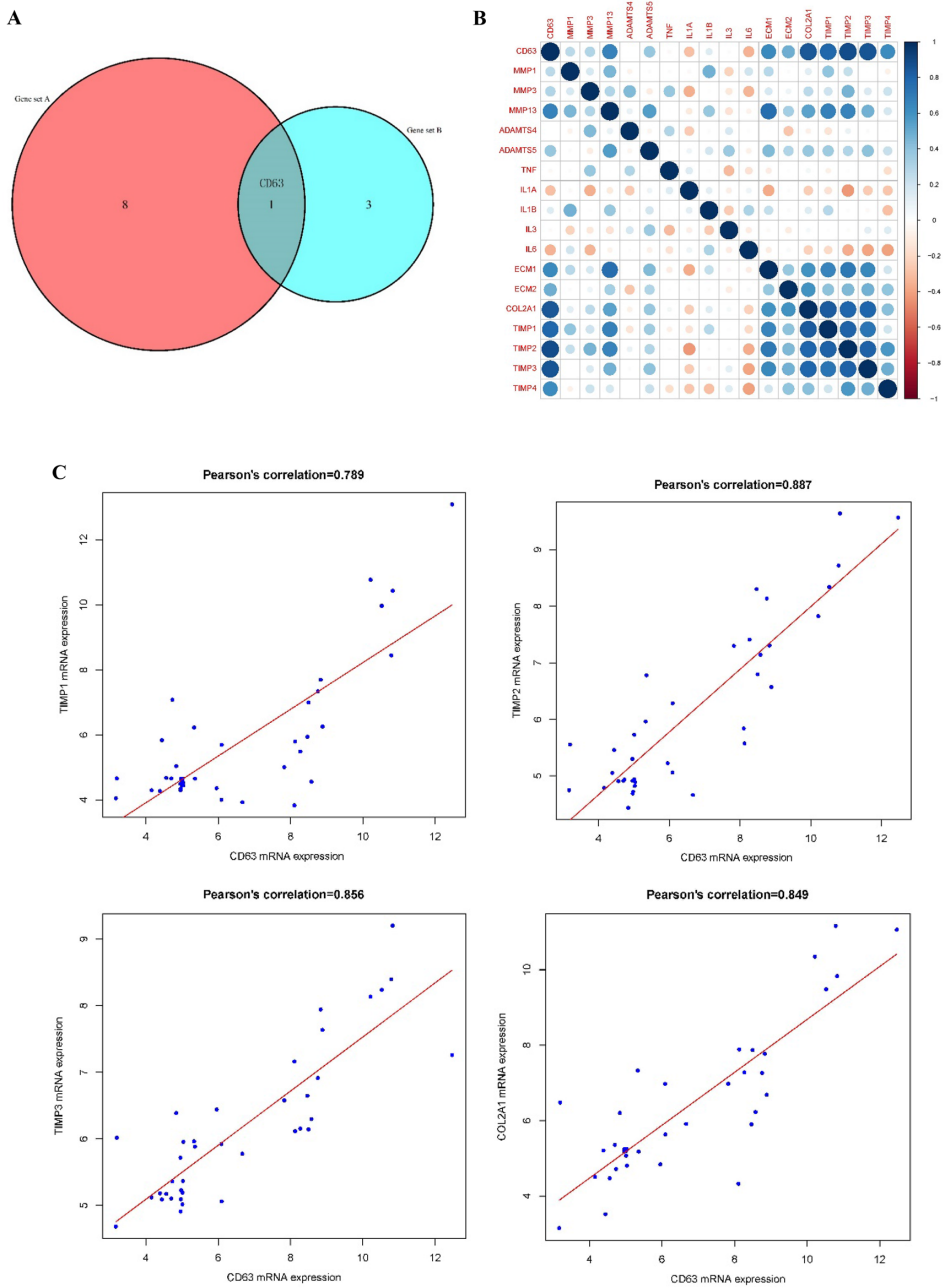
Selection of target genes. (**A**) The genes that closely related to intervertebral disc degeneration screened in weighted correlation network analysis (Gene set A) and co-expression network (Gene set B). (**B**) Pearson correlation analysis between CD63 and IL1, ECM, COL2A1, TIMP, MMP and ADAMTS. (**C**) Significant relatedness of CD63 with TIMP1, TIMP2, TIMP3 and COL2A1.

The genes that closely related to IDD, based on WGCNA (Gene set A) and Co-expression Network (Gene set B) screening were intersected and the hub gene CD63 obtained (Fig. 4A). Pearson Correlation Analysis between CD63 and IL1, ECM, COL2A1, TIMP, MMP and ADAMTS were performed (Fig. 4B). The results explored that CD63 was significantly related to TIMP1 (Cor = 0.789), TIMP2 (Cor = 0.887), TIMP3 (Cor = 0.856) and COL2A1 (Cor = 0.849) (Fig. 4C).

### GO, KEGG and GSEA

**Figure 5:**
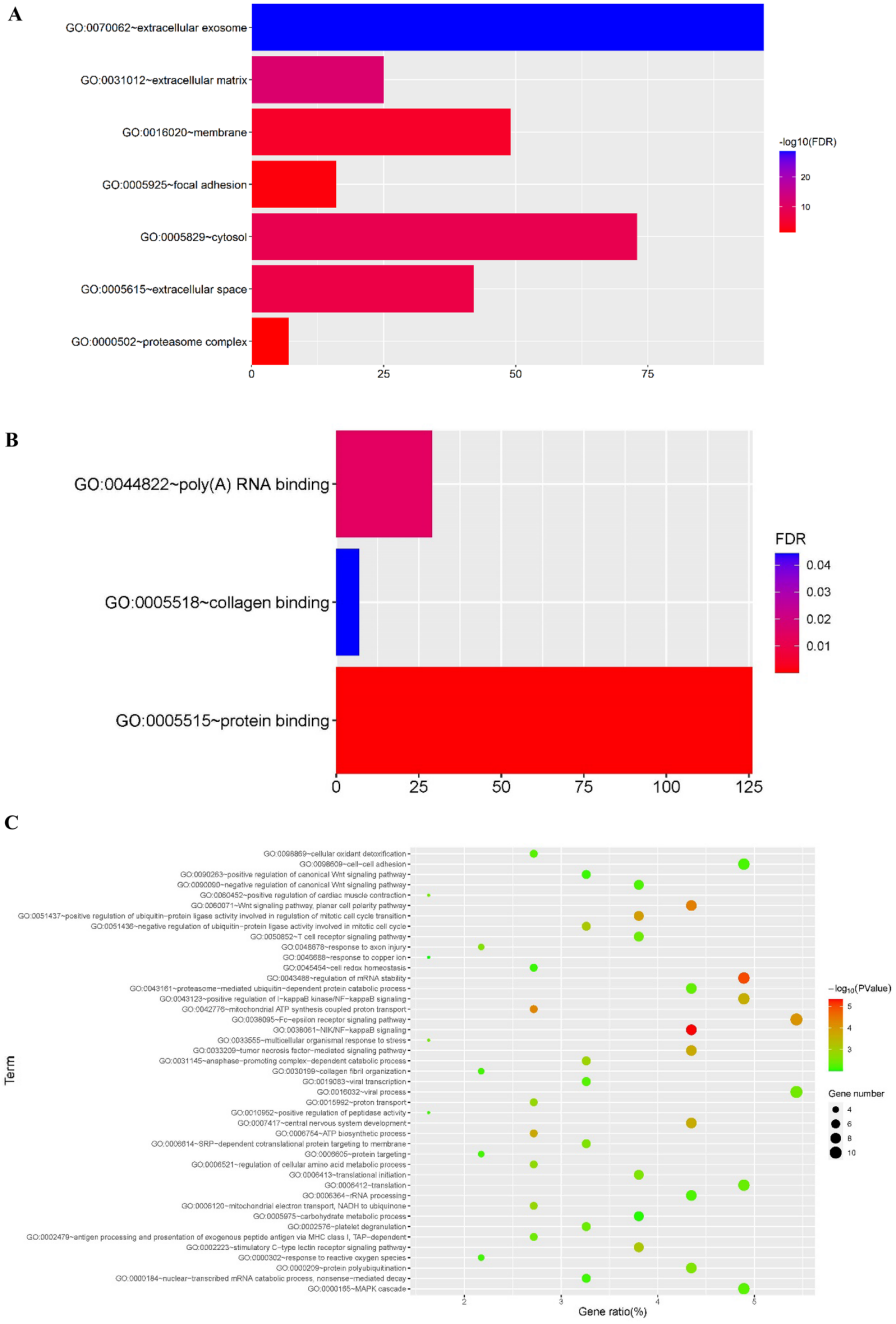
The analysis of GO (Cellular Component, Molecular Function and Biological Process) enrichment for intervertebral disc degeneration. (**A**) Genes in the hub module associated with intervertebral disc degeneration significantly enriched in 7 cellular components, (**B**) 3 items of molecular function and (**C**) 43 items of biological processes.

The analysis of GO (CC, MF and BP) enrichment for IDD was carried out. Genes in MEturquoise, the hub module associated with IDD, was significantly enriched in 7 cellular components (Fig. 5A), 3 items of molecular function (Fig. 5B) and 43 items of biological processes (Fig. 5C).

**Figure 6:**
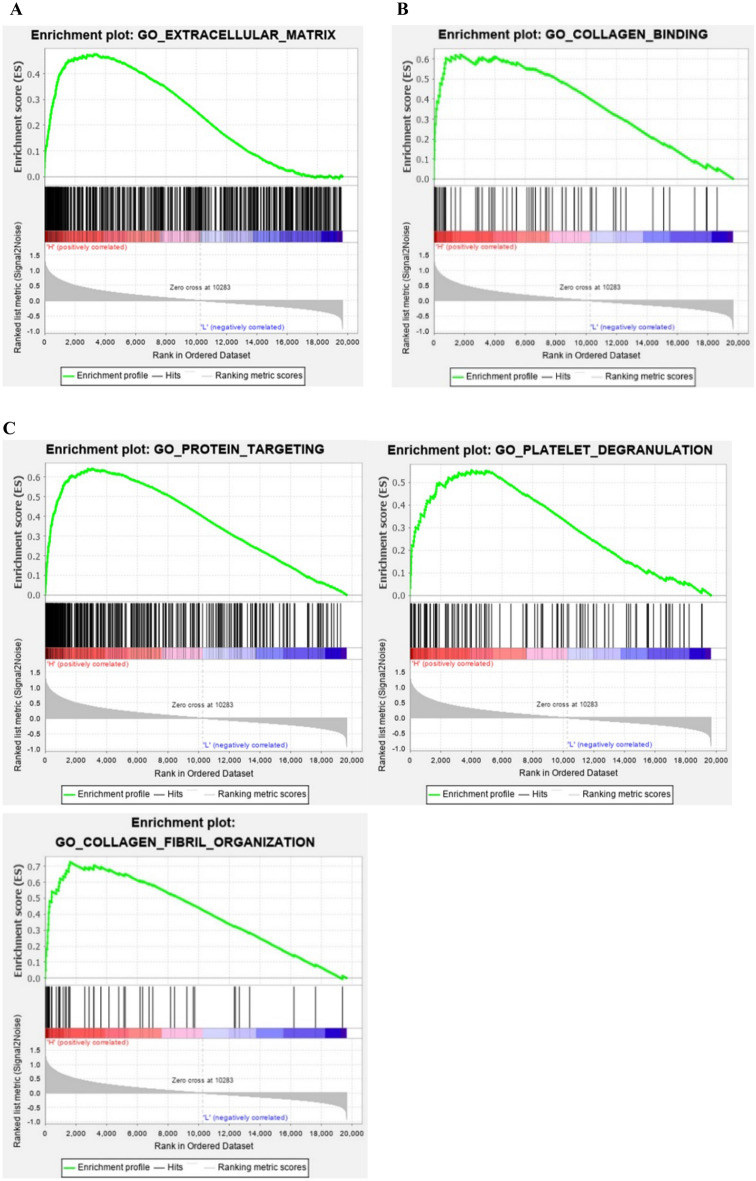
GO (cellular component, molecular function and biological process) related to intervertebral disc degeneration in the hub modules intersected with gene set enrichment analysis of CD63. The hub cellular component (extracellular matrix) (**A**) molecular function (collagen binding) (**B**) as well as three biological processes (protein targeting, collagen fibril organization and Platelet degranulation) (**C**) were finally obtained.

In the GSEA of CD63, 6 cellular components (|NES|> 1.8) (Table 4), 37 items of molecular function (|NES|> 1.78) (Table 5) and 42 items of biological processes (|NES|> 1.7) (Table 6) were obtained. Cellular components related to IDD that was screened from GSEA were intersected with that screened using WGCNA in hub module. The hub cellular component (Extracellular matrix) was finally obtained (Fig. 6A). The molecular functions and biological processes obtained from the GSEA of CD63 were intersected with that screened using WGCNA in the hub modules related to IDD. Finally, one hub molecular function (Collagen binding) (Fig. 6B) and three hub biological processes (Fig. 6C) were obtained.

**Table 4:** The GSEA of CD63 for cellular components.

Cellular components	P	FDR	NES
Extracellular exosome	< 0.001	0.0206	1.9779
Extracellular matrix	< 0.001	0.0932	1.8534
Cytosol	< 0.001	0.0777	1.8259
Extracellular space	0.0020	0.0641	1.8163
Membrane	< 0.001	0.0528	1.8147
Focal adhesion	0.0020	0.0529	1.8016

**Table 5:** The GSEA of CD63 for molecular function.

Term	P	FDR	NES
Extracellular matrix structural constituent	< 0.001	0.0654	1.9780
Translation elongation factor activity	< 0.001	0.0327	1.9761
WW domain binding	< 0.001	0.0232	1.9741
Fibroblast growth factor binding	< 0.001	0.0225	1.9589
Growth factor binding	< 0.001	0.0195	1.9501
Glycosaminoglycan binding	< 0.001	0.0383	1.8907
Protease binding	< 0.001	0.0397	1.8764
Disordered domain specific binding	< 0.001	0.0359	1.8720
Calcium dependent phospholipid binding	0.0021	0.0323	1.8695
RNA helicase activity	< 0.001	0.0372	1.8513
Beta catenin binding	< 0.001	0.0372	1.8452
Translation regulator activity	< 0.001	0.0359	1.8410
Cell adhesion molecule binding	< 0.001	0.0369	1.8351
SMAD binding	< 0.001	0.0347	1.8341
GTPase activity	< 0.001	0.0324	1.8335
Extracellular matrix structural constituent conferring compression resistance	0.0020	0.0306	1.8327
Ubiquitin like protein ligase binding	< 0.001	0.0297	1.8314
Kinase regulator activity	< 0.001	0.0293	1.8289
Copper ion binding	< 0.001	0.0312	1.8229
Translation regulator activity nucleic acid binding	< 0.001	0.0298	1.8217
Calcium dependent protein binding	< 0.001	0.0296	1.8196
ATPase binding	< 0.001	0.0283	1.8191
GDP binding	< 0.001	0.0262	1.8181
Ribosome binding	< 0.001	0.0274	1.8181
Hyaluronic acid binding	0.0021	0.0255	1.8176
Cadherin binding	< 0.001	0.0258	1.8137
Unfolded protein binding	< 0.001	0.0263	1.8082
Monosaccharide binding	< 0.001	0.0258	1.8080
mRNA binding	< 0.001	0.0252	1.8070
Heparin binding	< 0.001	0.0244	1.8066
Nucleobase containing compound transmembrane transporter activity	< 0.001	0.0252	1.8013
mRNA 3 UTR binding	< 0.001	0.0248	1.8002
Metalloendopeptidase inhibitor activity	0.0082	0.0255	1.7952
L ascorbic acid binding	< 0.001	0.0271	1.7904
Heat shock protein binding	< 0.001	0.0274	1.7877
Extracellular matrix structural constituent conferring tensile strength	0.0021	0.0270	1.7864
Collagen binding	0.0019	0.0274	1.7813

**Table 6:** The GSEA of CD63 for biological processes.

Term	P	FDR	NES
Keratan sulfate metabolic process	< 0.001	0.2063	1.9954
Cartilage development	0.0020	0.2377	1.7909
Protein targeting to membrane	0.0021	0.2327	1.7866
Pathway restricted SMAD protein phosphorylation	0.0020	0.2220	1.7857
Entrainment of circadian clock	< 0.001	0.2138	1.7840
Relaxation of cardiac muscle	< 0.001	0.2035	1.7837
Cellular response to gamma radiation	< 0.001	0.1974	1.7820
Chondroitin sulfate biosynthetic process	< 0.001	0.1929	1.7799
Proteoglycan biosynthetic process	0.0020	0.1965	1.7742
Artery development	< 0.001	0.1943	1.7705
Connective tissue development	< 0.001	0.1991	1.7647
Chondroitin sulfate proteoglycan biosynthetic process	0.0020	0.1938	1.7636
Negative regulation of cellular response to growth factor stimulus	< 0.001	0.1867	1.7635
RNA polyadenylation	< 0.001	0.1813	1.7630
Bone growth	< 0.001	0.1820	1.7599
Chondrocyte differentiation	0.0020	0.1785	1.7586
Aminoglycan metabolic process	0.0020	0.1850	1.7522
Labyrinthine layer blood vessel development	0.0021	0.1821	1.7504
Protein targeting	< 0.001	0.1774	1.7501
Regulation of morphogenesis of a branching structure	< 0.001	0.1742	1.7492
Positive regulation of morphogenesis of an epithelium	< 0.001	0.1709	1.7487
Growth plate cartilage morphogenesis	< 0.001	0.1794	1.7421
Negative regulation of transmembrane receptor protein serine threonine kinase signaling pathway	< 0.001	0.1748	1.7419
Sulfur compound biosynthetic process	< 0.001	0.1711	1.7413
Entrainment of circadian clock by photoperiod	< 0.001	0.1675	1.7409
Chondrocyte development	0.0020	0.1633	1.7408
Extracellular structure organization	< 0.001	0.1637	1.7378
Ovulation	< 0.001	0.1618	1.7369
Chaperone mediated protein folding	< 0.001	0.1667	1.7316
Regulation of protein maturation	0.0041	0.1630	1.7316
Sulfur compound catabolic process	0.0020	0.1678	1.7279
Chondroitin sulfate proteoglycan metabolic process	0.0020	0.1679	1.7260
Cell recognition	< 0.001	0.1694	1.7231
Bone development	< 0.001	0.1663	1.7229
Collagen fibril organization	0.0042	0.1640	1.7223
Regulation of DNA templated transcription in response to stress	< 0.001	0.1609	1.7222
Cellular aldehyde metabolic process	0.0021	0.1628	1.7191
Mesenchyme morphogenesis	< 0.001	0.1680	1.7146
Platelet degranulation	0.0020	0.1718	1.7114
Cranial nerve development	< 0.001	0.1783	1.7064
Aminoglycan catabolic process	0.0063	0.1782	1.7044
Regulation of receptor biosynthetic process	0.0021	0.1783	1.7025

### Signaling pathways

**Figure 7:**
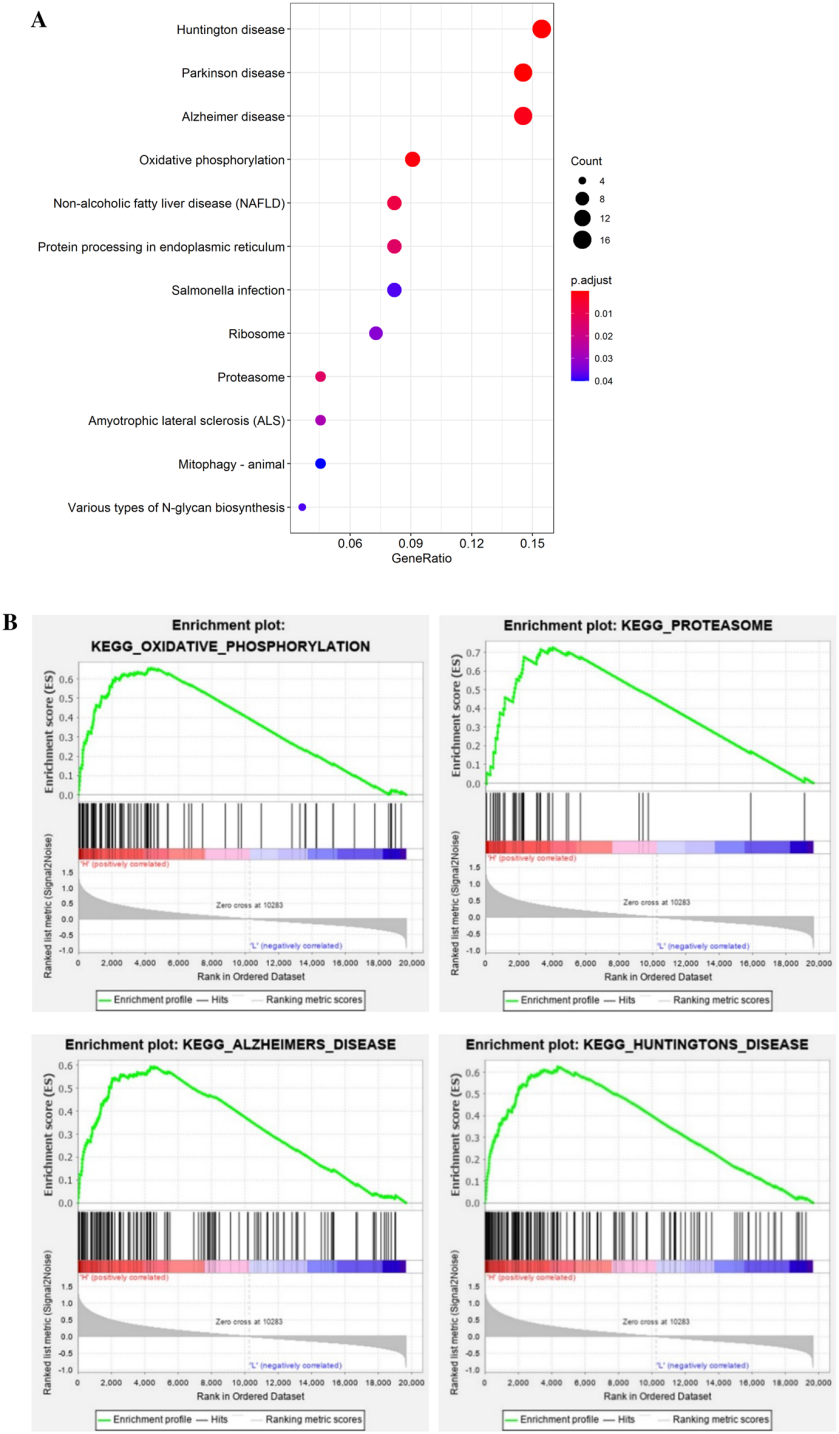
KEGG pathways related to intervertebral disc degeneration in the hub modules intersected with gene set enrichment analysis of CD63. (**A**) KEGG pathways significantly enriched in 12 signaling pathways. (**B**) 4 hub pathways were finally obtained when intersected KEGG pathways with gene set enrichment analysis of CD63.

The enrichment analysis of KEGG pathways were performed for the genes in hub module (MEturquoise) that were screened using WGCNA. Results showed that the genes were significantly enriched in 12 signaling pathways (Fig. 7A). In the GSEA of CD63, 20 items of signaling pathways (Table 7) (|NES|> 1.55) were enriched. The signaling pathways obtained from GSEA of CD63 were intersected with genes screened using WGCNA in the hub module related to IDD, and 4 hub signaling pathways (Fig. 7B) were finally obtained.

**Table 7:** The GSEA of CD63 for signaling pathways.

Term	P	FDR	NES
Cardiac muscle contraction	0.0079	0.0641	1.8305
Alzheimers disease	< 0.001	0.0676	1.7623
Huntingtons disease	< 0.001	0.0576	1.7500
Glycolysis gluconeogenesis	0.0039	0.0798	1.7115
Oxidative phosphorylation	< 0.001	0.0673	1.7080
Parkinsons disease	0.0020	0.0809	1.6816
Galactose metabolism	0.0078	0.0722	1.6767
Glycosaminoglycan biosynthesis chondroitin sulfate	0.0020	0.0801	1.6585
Selenoamino acid metabolism	0.0040	0.0834	1.6444
ECM receptor interaction	0.0020	0.0849	1.6342
Arrhythmogenic right ventricular cardiomyopathy	< 0.001	0.0873	1.6223
Spliceosome	< 0.001	0.0912	1.6101
Ribosome	0.0061	0.1003	1.5917
Glutathione metabolism	0.0059	0.1057	1.5809
N glycan biosynthesis	0.0040	0.1006	1.5790
Purine metabolism	0.0060	0.0948	1.5783
Focal adhesion	0.0059	0.0974	1.5704
Proteasome	< 0.001	0.1037	1.5548
Gap junction	0.0020	0.1033	1.5501
Notch signaling pathway	0.0079	0.0984	1.5501

### Expression of CD63 in human AF and NP tissues

**Figure 8:**
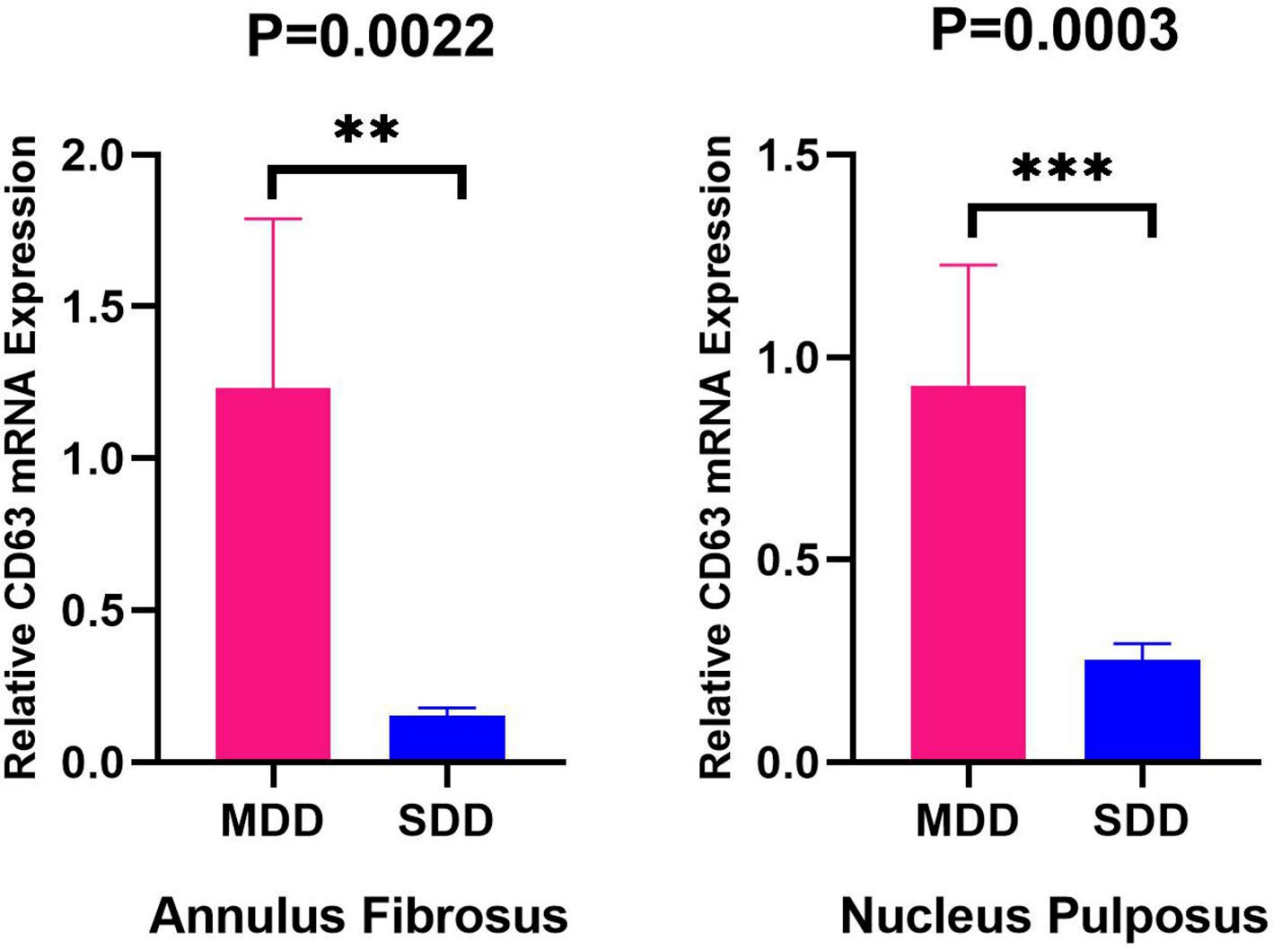
The expression of CD63 in annulus fibrosus and nucleus pulposus tissues from MDD (n = 6) and SDD (n = 6) patients with qRT-PCR. The mRNA expression of CD63 in annulus fibrosus and nucleus pulposus was markedly downregulated in SDD group compared to that in MDD group (P < 0.01).

To further study and validate the role of CD63 in different degrees of IDD, we measured the expression of CD63 in AF and NP tissues from MDD patients (n = 6) and SDD patients (n = 6) with qRT-PCR. Results showed that the mRNA expression of CD63 in AF and NP tissues was markedly downregulated in SDD group compared to that in MDD group (Fig. 8) (P < 0.01).

## Discussion

This study integrated 19,678 genes of 38 IDD patients from two gene datasets. We carried on multiple gene screening modes (DEGs Analysis, Pearson Correlation Analysis, WGCNA and Topological Characteristics) via biological information technology approach. Finally, the new regulatory gene for IDD, CD63, was identified.

CD63, also known as lysosome associated membrane protein 3 (LAMP3), belongs to the transmembrane 4 superfamily (TM4SF)^24–26^. The TM4SF members are related to each other and form a huge TM4SF network with some extra family proteins, which play vital roles in molecular metabolism^27^.

The gene CD63 can activate the surface antigens on platelets^28^. As a sensitive marker of platelet activation, detection of that index can sense the degree of platelet activation^29^, and then make a timely diagnosis and curative measure against the disease. Previous studies have reported the positive role of CD63 in the suppression of melanoma whereby it acted as a vital sign in patient assessment^30,31^. However, there is little research on the correlation between CD63 and IDD. Through multiple gene screening modes, we finally found the hub gene CD63. In the datasets, it was a significantly downregulated gene in SDD group compared to that in MDD group. Finally, the results of qRT-PCR of annulus fibrosus and nucleus pulposus tissues further validated that the expression of CD63 was markedly downregulated in SDD group. Different degrees of IDD may be closely related to the reduced expression of CD63. It may become a new promising gene for IDD and help us further enrich the therapeutic targets of IDD.

Extracellular matrix (ECM) located outside one or more cells and provided structural support. The main components of ECM are proteoglycans and collagen II that maintain the physiological function and stability of IVD. The components are reduced during the process of IDD, thus can be used as an important sign for IDD^32^. The ECM plays critical role in the maintenance of steady state for IVD and different degrees of IDD. CD63 was a cell surface binding partner for Tissue Inhibitor of Metalloproteinases-1 (TIMP-1) which play important role in the degradation and synthesis of matrix^33^.

The result of GO enrichment analysis also showed that CD63 play an important role in cell matrix. This is consistent with previous studies. It suggested that CD63 may participate in the regulation of IDD through ECM.

The screened MF of CD63 obtained using the conjoint analysis of WGCNA and GSEA were "collagen binding". Collagen, a main component of IVD, was fully demonstrated in IDD^34^. Collagen binding implies that a group of fibrous proteins with high tensile strength interact with collagen selectively and non-covalently. The Pearson Correlation Analysis further indicated that CD63 was significantly related with TIMP and collagen (Fig. 4). Previous studies had explored that TIMP and collagen were closely related to IDD, and both were important indicators of IDD^16,17^. Takawale et al^35^ identified a novel mechanism in vivo for TIMP1, CD63 and collagen synthesis, as well as CD63 shown to exist as a cell surface receptor for TIMP1. Their study also showed that TIMP1 mediates an association between CD63 and integrin β1, leading to de novo collagen synthesis on cardiac fibroblasts. The screened MF of CD63 (Collagen binding) explored potential molecular mechanism of CD63, TIMP1 and collagen in different degrees of IDD. Using yeast two-hybrid screening, Jung et al. identified CD63 as a cell surface binding partner for TIMP1 which played a critical role in TIMP1-mediated cell survival signaling and apoptosis inhibition^33^. In the future, the relationship between CD63 and TIMP1 would be reported in more diseases.

In addition, the significant enrichment of “protein targeting” and “collagen fibril organization” in BP, as well as “proteasome” and “oxidative phosphorylation” in the pathway of CD63 explored new basis and implications for the study of CD63 in IDD. Many previous studies also have reported the causes of IDD for example, decreased expression of collagen II and proteoglycan, increased expression of enzymic degradation in ECM and apoptosis in nucleus pulposus cells^36–38^.

Although we have tested the predictive results through clinical samples and explored their possible mechanism, the sample sizes may be still insufficient according to the difference between mild and severe disc degeneration groups studied with bioinformatic analysis. To the best of our knowledge, similar reports are rare. Therefore, in prospective studies, we intend to analyze the underlying molecular mechanisms mechanism of CD63 for IDD with multiple biological methods.

## Conclusion

Our study explored and validated a new target gene CD63 for different degrees of IDD. The study and its findings are important as they form a basis upon which new therapeutic targets for IDD can be identified.

## Supplementary Information

Supplementary Information.

## Data Availability

The two available datasets in this paper are from the Gene Expression Omnibus (GEO) database (https://www.ncbi.nlm.nih.gov/geo/).
