# Supplementary material for: A potential target gene CD63 for different degrees of intervertebral disc degeneration
Source: Sci Rep. 2022 Jan 19;12:957. doi: 10.1038/s41598-022-05021-4 (PMC8770635; doi:10.1038/s41598-022-05021-4)
Supplement: Supplementary file 1 — Supplementary Information. [file 41598_2022_5021_MOESM1_ESM.docx]

**A B C D**


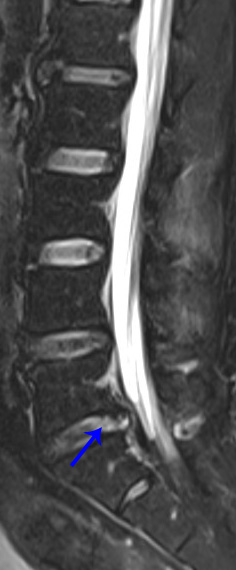

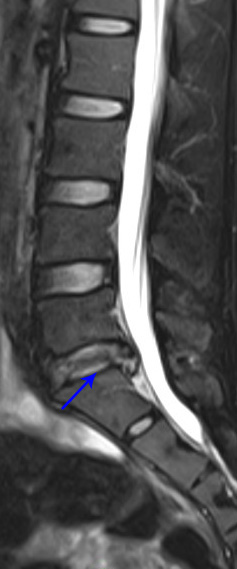

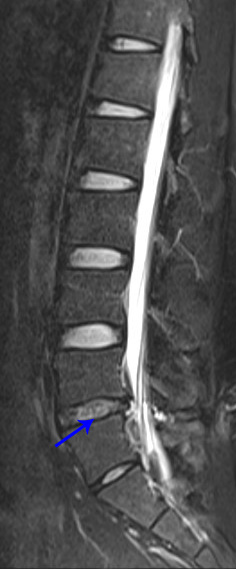

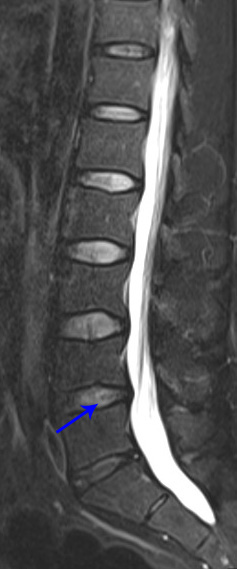


**E F**


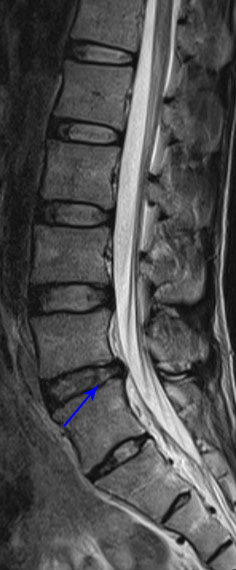

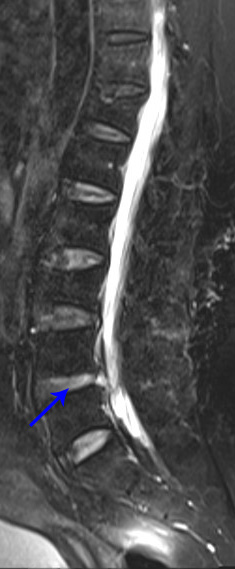


**Supplementary Figure 1 | (A-F)** The MRI of spine for MDD patients, and the surgery segment of lumbar discectomy **(Blue arrow)**.

**A B C D**


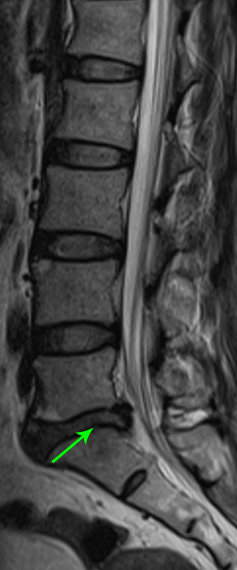

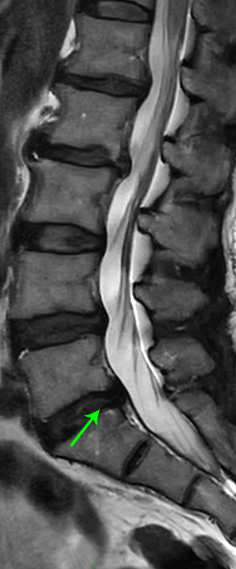

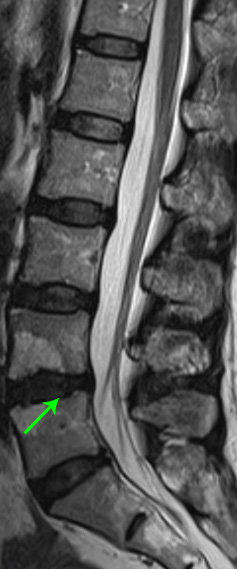

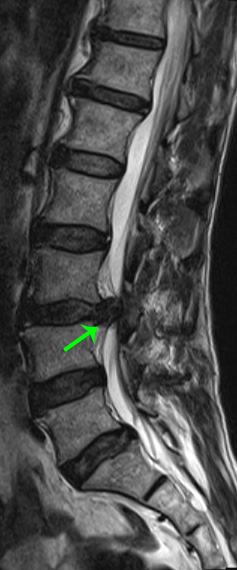


**E F**


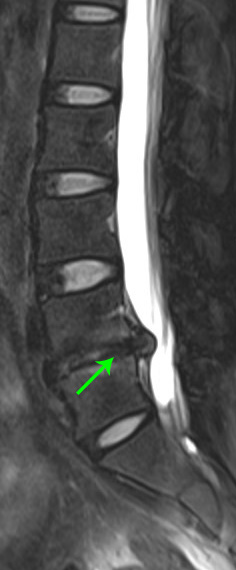

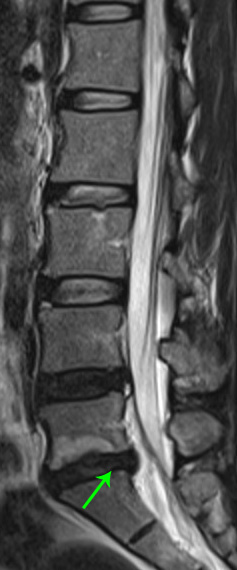


**Supplementary Figure 2 | (A-F)** The MRI of spine for SDD patients, and the surgery segment of lumbar discectomy **(Green arrow)**.
